# A Unique Case of Unilateral Lower Extremity Sparing Systemic Peripheral Gangrene

**DOI:** 10.1155/2012/750953

**Published:** 2012-12-13

**Authors:** Alexey Markelov, Steven DeFroda, Leopoldo Baccaro, Jamie Bastidas

**Affiliations:** ^1^Department of Surgery, Easton Hospital, Drexel University College of Medicine, Easton, PA 18042, USA; ^2^Drexel University College of Medicine, Philadelphia, PA 19129, USA

## Abstract

We present here the case of a 70-year-old female who developed a systemic peripheral gangrene in both of her upper extremities (all fingers) and her right foot due to a severe septic shock requiring a systemic vasopressor therapy. Interestingly, the patient's left foot remained spared from gangrenous changes possibly due to a chronic external iliac artery occlusion and thus the lower concentration of vasopressors in that extremity.

## 1. Introduction

Systemic peripheral gangrene (SPG) is defined as a symmetric ischemia in the absence of a large vessel obstruction in at least two of the following: distal extremities, tip of the nose, ears, and genitals [1, 2]. It typically occurs in the face of an infectious disease or low flow states such as cardiogenic shock [3]. It can also occur as a result of vascular problems such as Reynaud's, c-protein deficiency, sickle cell disease, and paraneoplastic syndromes [4]. Iatrogenic causes such as the administration of systemic vasopressor in the presence or absence of sepsis have also been shown to cause SPG. Our patient developed gangrenous necrosis in all of the extremities except the left foot. This was a curious finding; however, further investigation revealed that the patient had a chronic occlusion of the left superficial femoral artery. It was hypothesized at the time, and currently believed, that this significant obstruction prevented the “adequate” delivery of vasopressors to her distal lower extremity, preventing excessive vasospasm and subsequent necrosis. A review of the literature does not reveal any similar cases of a patient experiencing SPG with one limb spared secondary to underlying undiagnosed peripheral artery disease.

## 2. Case Report

**Figure 1:**
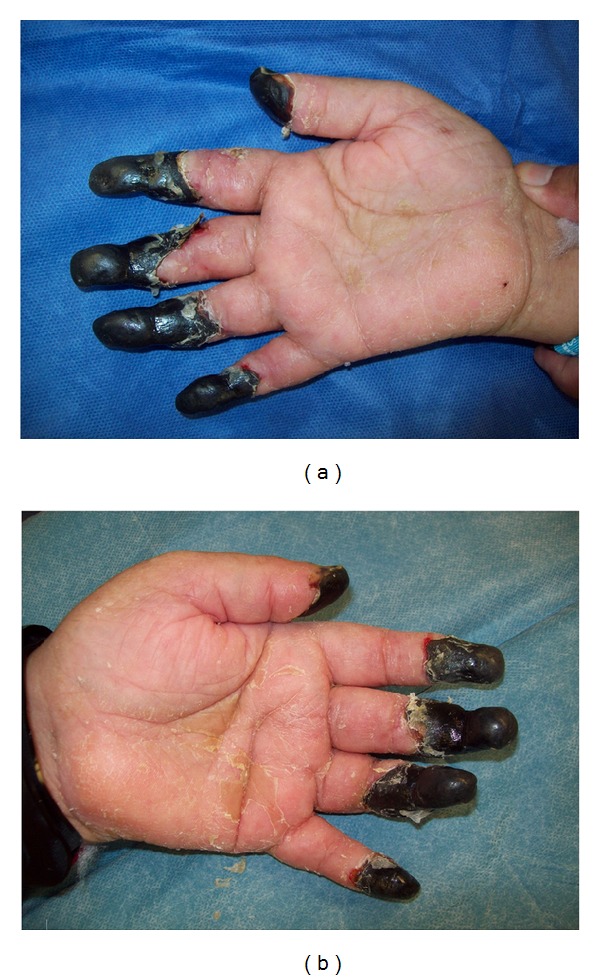
Bilateral peripheral gangrene of upper extremities.

70-year-old Hispanic female with the past medical history of congenital paralysis below the waste presented with an episode of urinary tract infection caused by *E. coli*. The patient subsequently developed a septic shock with multi-system organ failure and disseminated intravascular coagulation that required a vasopressor therapy. The patient required an administration of high doses (up to 30 mcg/kg/min) of norepinephrine in conjunction with vasopressin during prolonged ICU course. Secondary to that the patient developed a bilateral dry gangrene of all distal phalanges, as well as the gangrene of the distal right foot from tarsal to the metatarsal level (Figure 1).

**Figure 2:**
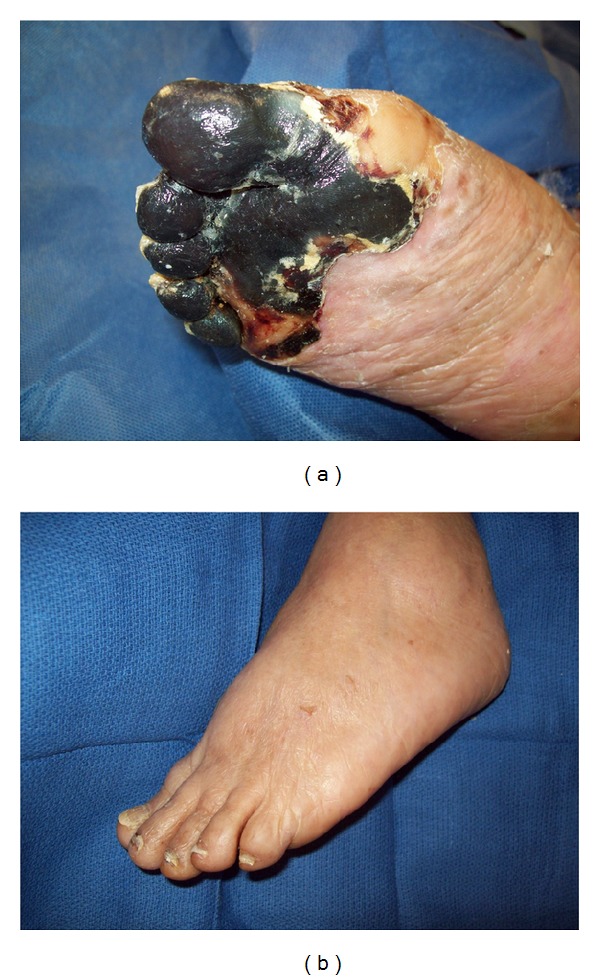
Unilateral lower extremity sparing systemic peripheral gangrene.

The left foot, however, remained spared from any ischemic changes (Figure 2).

**Figure 3:**
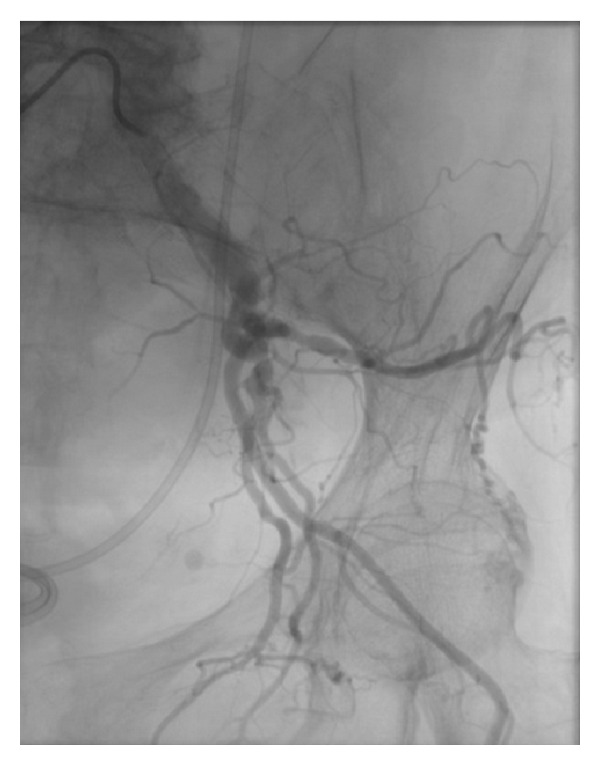
Left lower extremity arteriogram showing the chronic occlusion of external iliac artery.

Subsequent cardiac catheterization study incidentally revealed the chronic occlusion of the left external iliac artery (Figure 3), which most likely prevented the infarct of the left lower extremity. The patient eventually underwent bilateral amputations of all fingers and toe amputations on her right lower extremity.

## 3. Discussion

Symmetrical peripheral gangrene (SPG) is a well-documented but rare clinical syndrome characterized by symmetrical distal ischemic damage leading to gangrene of two or more sites in the absence of a large vessel obstruction or vasculitis. Hutchison first described SPG in 1891 [3]. Although described more than a century ago, most of the cases of SPG have been documented as single case reports. A review of English language medical literature that we conducted did not reveal any similar cases of patient experiencing SPG with one limb spared secondary to underlying undiagnosed peripheral artery disease.

A wide array of infective and noninfective etiological factors has been linked with the development of SPG (Table 1).

The exact pathophysiological mechanism of the vascular occlusion in SPG is uncertain. A low-flow state along with disseminated intravascular coagulation (DIC) is usually present. DIC is an almost universal finding and is probably the final common event in the microvascular insult that gives rise to the typical clinical features of this syndrome. Diagnosis and management of DIC should be guided by basic tests to evaluate the coagulation cascade (PT, PTT, and fibrin split products) and if found, it should be treated early and aggressively. Identification and treatment of the underlying cause is the most important part of the treatment. It is important to remember that vasopressor agents, commonly used in the management of sepsis-induced hypotension, may aggravate SPG.

In conclusion, SPG is a cause of significant morbidity and mortality often requiring multiple limb amputations in the survivors. Thus, early recognition of SPG and its underlying conditions can have a profound impact on the management of the condition and its final outcome.

## Figures and Tables

**Table 1:** Causes of systemic peripheral gangrene.

Causes of SPG			
Infectious	Pharmacologic		Systemic
Bacterial sepsis	Dopamine	Diltiazem	Paraneoplastic syndrome
Viral sepsis	Inotropin	Haloperidol	Ergotism
Rickettsia	Epoprostenol	Thorazine	Polymyalgia rheumatica
	Phenoxybenzamine	Cocaine	Raynaud's phenomenon
	Phentolamine	Amphetamine	C protein deficiency
	Trimethaphan	Thiopentone	Sickle cell disease
